# Influence of Graphene Nanoplatelet Lateral Size on the Electrical Conductivity and Electromagnetic Interference Shielding Performance of Polyester Nanocomposites

**DOI:** 10.3390/polym13152567

**Published:** 2021-07-31

**Authors:** Milad Madinehei, Scheyla Kuester, Tatiana Kaydanova, Nima Moghimian, Éric David

**Affiliations:** 1 Mechanical Engineering Department, École de Technologie Supérieure, 1100 Notre-Dame St W, Montréal, QC H3C 1K3, Canada; miladmadinehei@gmail.com; 2 NanoXplore Inc., 4500 Boulevard Thimens, Saint-Laurent, QC H4R 2P2, Canada; Scheyla.Kuester@nanoxplore.ca (S.K.); Tatiana.Kaydanova@nanoxplore.ca (T.K.); nima.moghimian@nanoxplore.ca (N.M.)

**Keywords:** nanocomposite, graphene, EMI shielding, electrical properties, absorption, polyester

## Abstract

Polyester nanocomposites reinforced with graphene nanoplatelets (GnPs) with two different lateral sizes are prepared by high shear mixing, followed by compression molding. The effects of the size and concentration of GnP, as well as of the processing method, on the electrical conductivity and electromagnetic interference (EMI) shielding behavior of these nanocomposites are experimentally investigated. The in-plane electrical conductivity of the nanocomposites with larger-size GnPs is approximately one order of magnitude higher than the cross-plane volume conductivity. According to the SEM images, the compression-induced alignments of GnPs is found to be responsible for this anisotropic behavior. The orientation of the small size GnPs in the composite is not influenced by the compression process as strongly, and consequently, the electrical conductivity of these nanocomposites exhibits only a slight anisotropy. The maximum EMI shielding effectiveness (SE) of 27 dB (reduction of 99.8% of the incident radiation) is achieved at 25 wt.% of the smaller-size GnP loading. Experimental results show that the EMI shielding mechanism of these composites has a strong dependency on the lateral dimension of GnPs. The non-aligned smaller-size GnPs are leveraged to obtain a relatively high absorption coefficient (≈40%). This absorption coefficient is superior to the existing single-filler bulk polymer composite with a similar thickness.

## 1. Introduction

The rapid development of electronic technologies and devices has increased concerns regarding electromagnetic (EM) pollution in the microwave range. The radiated EM waves can not only treat human health, but might also interfere with other electronic equipment and affect their normal operation [1,2]. The standard technical method for minimizing EM pollution employs electromagnetic interference (EMI) shielding materials to isolate the electronics from the surrounding environment. Traditional EMI shielding methods utilize the EM wave shielding properties of metals such as nickel, aluminum, copper etc., [3,4]. These materials, characterized by high electrical conductivity (∼10^5^ S/cm), attenuate EM waves owing to strong skin effects. The performance of shielding materials is mainly a function of their ability to reflect and absorb undesirable waves, with EM waves absorption being the preferred quality. Although metallic shields possess high EMI shielding efficiency (SE), their shallow penetration depth (skin depth in the order of 1 micron or less) limits their ability to absorb EM waves. Moreover, the additional weight of metal-based materials, coupled with their susceptibility to corrosion, makes them less desirable in shielding applications.

Owing to their superior qualities, such as light weight, good processability, good environmental stability, and tunable morphology [5,6,7], conductive polymer nanocomposites have been explored as an alternative to metal shields for the last 10 years. Conductive polymer composites are the multi-phase composites obtained by adding electrical nanofillers (such as graphene, carbon nanotube, MXenes, etc.,) into the polymer matrix using specific processing technologies. A wide range of EMI SE values for different composites have been reported in the literature depending largely on the processing parameters, polymer matrix, and nanofiller type [8,9,10,11,12,13,14]. While significant advances have been made in understanding the benefits of these electroconductive composites, there continue to be certain challenges, which have inhibited the widespread application of these material in an EMI environment. Despite demonstrating an acceptable shielding performance (SE > 20 dB), other metrics such as processibility and the cost of raw materials have not been considered in most related studies. For example, the outstanding electrical conductivity and chemical activity of newly emerged MXenes (2D transition metal carbides or nitrides) could result in an ultraefficient EMI SE of over 40 dB in the gigahertz (GHz) region [9,15]. However, at the present time, the cost of MXenes is relatively high, and the preparation process is more complicated. Even a moderate EMI SE (over 20 dB) requires high nanoparticle loading (5–40 wt.%), which inevitably leads to low affordability. Carbon nanotube (CNT) which is known to have exceptionally high electrical conductivity (10^5^ S/cm), can be used in applications requiring high level of EMI attenuation; for example, a SE of 50 dB was reported for 15 wt.% CNT/ABS nanocomposite [7,8]. Nevertheless, a primary drawback for applications of CNTs has been their cost, specially, if single-walled CNT is warranted. Similarly, most of the fillers used from graphene family, are economically non-viable, difficult to produce at bulk scale and often require purification, auxiliary treatment, and functionalization steps [6,7]. As an example, despite achieving high SE by reduced graphene oxide, transferring these scientific findings to industry has been hindered by the cost and the lack of a large-scale reduction method. A low-cost EM shield with a simple and scalable production method thus still needs to be developed for industrial applications.

On the other hand, compared to the reflection loss dominant mechanism, which may cause new reflected wavefront (secondary pollution), EM wave absorption represents a more efficient way to shield EM waves and reduce undesirable emissions. However, realizing highly efficient EMI shielding with microwave absorption-dominated features remains a tough challenge. Absorption mainly occurs inside the shield, which can convert the EM energy into thermal or other forms of energy through dielectric/magnetic loss [7,8,9]. The dielectric or magnetic loss capacity of shielding materials can be enhanced by adding different kinds of magnetic or electric nanofillers, but unilaterally increasing the permittivity or permeability could result in impedance (ratio between the electric and magnetic fields) mismatch, and in an increase in incident wave reflections. A comprehensive literature review on the absorption versus the reflection of bulk conductive nanocomposites has revealed that most such materials directly generate significant EM wave reflections on their surfaces, where the reflected power contributes to at least 80% of the total shielding efficiency [6,7].

Recently, graphene nanoplatelets (GnPs) have been identified as promising candidates for incorporation into polymeric matrices since they are typically less expensive than other forms of graphene. Beside the versatility of GnP [16,17,18], its production has also been scaled up to provide ton-scale quantities. The electrical properties of GnPs are comparable to those of many carbonaceous nanofillers [19,20] and they exhibit good compatibility with several polymers. For example, epoxy [21], polyester [22], HDPE [23] lactide (PLA) [24], poly (lactic acid) [25], and poly (vinylidene fluoride) (PVDF) [26] have been used as host media for GnP, and their electrical properties have been reported.

In this study, polyester composites reinforced with two different types of GnPs were prepared and the EMI-shielding behavior of each was examined and compared. This nanocomposite is very promising for use as an effective and practical EMI shielding material owing to its adequate shielding efficiency, low cost, and easy processability. In the study, the separate contributions of reflection and absorption loss towards the overall EM attenuation of the composites are illustrated. The composite with better absorption features is determined, and a possible absorption mechanism is proposed. To correlate the EMI behavior of this composites with electrical characteristics, the electrical conductivity of samples was measured in two (x-y) directions. During its service life, an EMI shielding material may be subject to high temperature. Therefore, the effect of GnPs on the thermal stability of the matrix is also explored.

## 2. Materials and Methods

### 2.1. Materials

Two commercially available GnPs were supplied by NanoXplore Inc. (Canada), GrapheneBlack^TM^3X and GrapheneBlack^TM^0X. These materials are respectively referred to as Gr3x and Gr0x herein. According to the supplier datasheet, these GnPs have a similar average thickness, which corresponds to 6–10 graphene monolayers, and average lateral dimensions of ~38 µm and ~13 µm, respectively. These two grades of GnPs are mass-produced at a scale of several tons per year via the mechanochemical exfoliation of natural graphite, which is a water-based, environmentally friendly technique. 

The polymer matrix used to generate the composites loaded with Gr3x or Gr0x was a non-air inhibited polyester resin, H596-HWA-15, with a viscosity of 600 cp (measured with LV at 60 RPM), which was purchased from AOC Company (Guelph, ON, Canada). 

High surface energy of graphene leads to strong tendency of these nanoparticles to form agglomerates. High shear mixing is known to be a good tool for the de-aggregation and dispersion of nanofillers into thermosetting resins. GnP/polyester nanocomposites were prepared by mixing polyester resin and GnPs (5–25 wt.%) in a high shear mixer at high speed (2000–10,000 RPM) for 5–10 min. The hardener (methyl ethyl ketone peroxide [MEKP]) was subsequently added at a concentration of 2 wt.% of the entire composite weight. After an additional 2 min of hand mixing, the mixture was poured into disk-shaped Teflon molds (diameter, 40 mm; thickness, 3.0 mm) and cured with a hot press machine for 2 h at 70 °C under 5 MPa. The samples were subsequently post-cured for an additional 2 h at room temperature.

### 2.2. Morphological Characterization

The transmission electron microscope (TEM) images of the GnPs were taken with a JEOL TEM 2100-F 200 kV field emission gun microscope. The dispersion state of the GnPs in the (cured) polyester nanocomposites was investigated using a SU-8230 Hitachi scanning electron microscope (SEM) operated at 5 kV. Samples were coated with gold using a sputtering coater before the morphology was observed.

### 2.3. Thermogravimetric Analysis (TGA)

The thermal stability of the samples was investigated using a thermogravimetric analyzer (Model Q500, TA Instruments). Samples weighing approximately 5 mg were used for each experiment. The TGA was operated in air and the heating rate was set to 10 °C/min, from 30 to 1000 °C.

### 2.4. Electrical Characterization

The in-plane direct current (dc) conductivity of the GnP/polyester nanocomposites was measured using a home-made Four Point Probe set-up with a needle spacing of 2.6 mm. A Keithley 237 instrument was used to pass a known current through the two outer probes, while an Agilent 3458A voltmeter measured the output voltage across the inner probes.

To characterize the cross-plane electrical conductivity of the composites, broadband dielectric spectroscopy (BDS) was performed within the 10^−2^ Hz to 10^+6^ Hz frequency range. Measurements were made with an Alpha-A Frequency Response Analyzer (Novocontrol Technologies). The frequency analyzer applied 3 V_RMS_ to each sample through the sample holders and measured the complex capacitance, $C^{*}=C_{o}\varepsilon^{*}$, with $C_{o}$ being the empty cell/air capacitance and $\varepsilon^{*}=\varepsilon^{\prime }-i\left(\varepsilon^{\prime \prime }+\frac{\sigma }{\omega \varepsilon_{o}}\right)$ and $\varepsilon^{\prime }$ and $\varepsilon^{\prime \prime }$ being the real and imaginary parts of the complex permittivity, respectively. The complex conductivity, $\sigma^{*}$, can be defined as $\sigma^{*}=i\varepsilon_{o}\omega \varepsilon^{*},$ where $\sigma^{\prime }\left(\omega \right)=\omega \varepsilon_{o}\varepsilon^{\prime \prime }\left(\omega \right)+\sigma$ and $\sigma^{\prime \prime }\left(\omega \right)=\omega \varepsilon_{o}\varepsilon^{\prime }\left(\omega \right)$, ω is the angular frequency, and $\varepsilon_{o}$ is the permittivity of free space. The real part of the complex conductivity represents both ohmic losses due to the flow of free charge carriers and the frequency-dependent dielectric losses. As can be seen from the expression above, at low frequencies, the contribution of the dielectric losses to the real part of the complex conductivity is greatly diminished, and therefore, the $\sigma^{\prime }$ measured is dominated by free-charge-carrier “dc” conductivity. Consequently, in the present work, the cross-plane dc conductivity value was assumed to be equal to $\sigma^{\prime }$ measured at the low frequency of 10^−1^ Hz.

### 2.5. EMI Measurements

When an EM wave propagating in free space encounters a shield, a part of an incident wave is attenuated through reflection and absorption mechanisms, and the rest of the wave is transmitted.

In the present study, EM scattering (*S*) parameters, which define the shielding efficiency of the material in terms of reflected power (*R*) and transmitted power (*T*), were measured using a Keysight E5063A ENA 2-port Vector Network Analyzer over an 8 GHz to 13 GHz (X-band) frequency range according to the waveguide method [3,7]:

(1) $$R={\left|S_{11}\right|}^{2}$$

And

(2) $$T={\left|S_{21}\right|}^{2}$$

where *S*_11_ corresponds to the voltage reflection coefficient of port 1, and *S*_21_ is the ratio of the outgoing wave voltage amplitude coming out of port 2, to the incident wave voltage amplitude at port 1. Subsequently, absorbed power, *A*, can be obtained from the simple relation: (*A* + *R* + *T*) = *I,* where *I* is the power incident on the shielding material. The total shielding efficiency (*SE*_tot_) of a material is the sum of its shielding efficiency due to reflection and absorption mechanisms, and is defined as the ratio between the incoming power (*I*) and transmitted power (*T*) of an EM wave:

(3) $$SE_{tot}=10\log\left(\frac{I}{T}\right),\;$$

with the unit of decibel (dB).

## 3. Results

### 3.1. Electrical Conductivity

**Figure 1:**
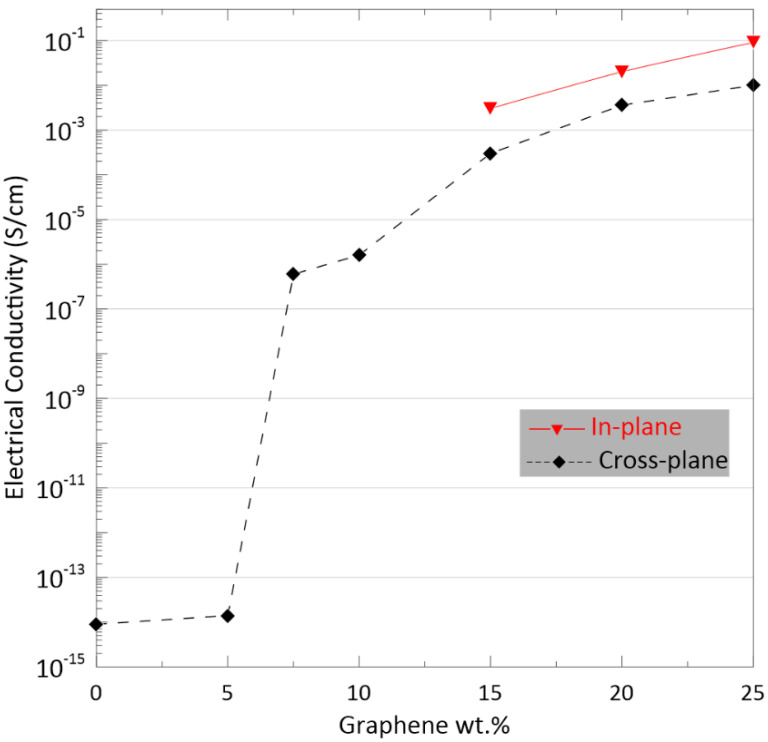
Electrical conductivity versus the GnP content for the Gr3x/polyester composites.

The variation of the dc conductivity of polyester nanocomposites as a function of Gr3x loading is plotted in Figure 1. The cross-plane conductivity ($\sigma_{cross}$) of neat polyester and composite with the low loading fraction *ϕ* = 5 wt.% were determined to be 9 × 10^−15^ and 1.4 × 10^−14^ S/cm, respectively. At low concentrations, conductivity is achieved via electron tunneling, which occurs through the interfaces that are formed between the filler and the host matrix [27]. Since the bound charges of the polyester belong to the valence band, the entire composite exhibits an insulative behavior. By further increasing the GnP fraction, the fillers get closer together, and ultimately, at a critical concentration range called the percolation threshold, the first conductive networks form. The conductivity determined for the nanocomposite loaded with 7.5 wt.% GnPs significantly increased to 6 × 10^−7^ S/cm, which is almost 7 orders of magnitude higher than that of the composite loaded with 5 wt.% Gnps.

Within the conductive region (*ϕ* > 7.5 wt.%), as the concentration of filler increases, the available free electrons in the composite will increasingly play the role of charge carriers due to the formation of a conductive network inside the sample. At the highest concentration of GnP loading tested (*ϕ* = 25 wt.%), the conductivity of the composite reached a value of 1 × 10^−2^ S/cm, which implies that a well-developed three-dimensional (3D) conductive network was formed.

The electrical properties of polymer nanocomposites depend on the intrinsic properties of fillers, as well as on some other parameters, such as filler–filler interactions, dispersion, distribution, and orientation of nanofiller particles [28,29]. Consequently, simply adding conductive fillers does not guarantee a conductive composite, and a uniform dispersion of conductive fillers is still required for the coherent movement of charges (conductivity) in a composite.

**Figure 2:**
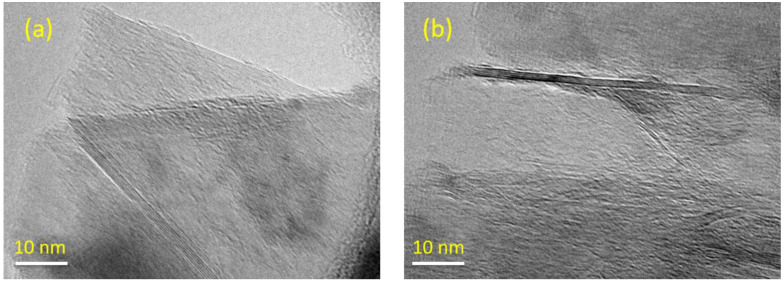
TEM images of Gr0X (**a**) and Gr3X (**b**).

To investigate the dispersion and distribution state of GnPs in the polyester matrix, SEM images were taken from the fracture surface of the composite samples. TEM was also employed to characterize the morphology of the GnPs. Figure 2a,b respectively represent typical HR-TEM images of Gr0X and Gr3X individual platelets. In both particles, the stack-like morphology (a typical structure for GnPs) can be clearly observed. The thickness of the GnPs is <3 nm (correspond approximately to 10 graphene layers), which is in a good agreement with the technical datasheet provided by the supplier.

**Figure 3:**
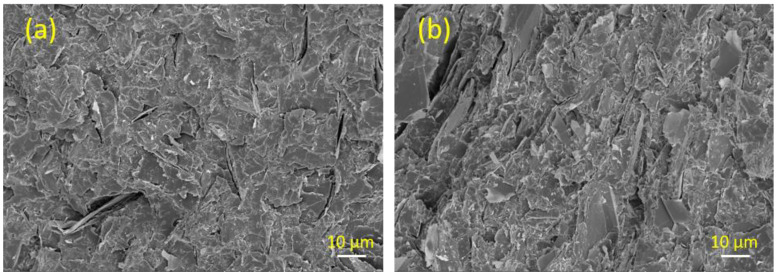
SEM images of cross-sections of GnP/polyester composites containing (**a**) 7.5 wt.% Gr3x versus (**b**) 25 wt.% Gr3x.

The high quality of the dispersion that was achieved by shear mixing of the GnPs in the polyester matrices can be seen on the SEM images of Figure 3.

A cross-section of the GnP/polyester composite prepared with 7.5 wt.% Gr3x loading is shown in Figure 3a. The uniform distribution and overlapping of the filler particles that can be observed for the 7.5 wt% GnP polyester composite confirm that this level of Gr3X loading exceeds the percolation threshold. As shown in Figure 3b, the composites with 25 wt.% Gr3X loadings contain aggregates of GnP that are well distributed and adjacent to each other, implying that a continuous interconnected network has been formed throughout the matrix.

For moderate and high resistivity, the power supply used by the four-probe test set-up cannot provide sufficient voltage to maintain a measurable current flow. Therefore, the in-plane conductivity $\left(\sigma_{in}\right)\;$ was only measured for the samples with conductivity values greater than 10^−3^ S/cm. The $\sigma_{in}$ data as presented in Figure 1 show that the $\sigma_{in}$, which mainly affects the EMI reflection mechanism, was approximately one order of magnitude higher than the $\sigma_{cross}$ of these composites. For the sample containing 25 wt.% of Gr3x, $\sigma_{in\;}$ reached 2 × 10^−1^ S/cm.

**Figure 4:**
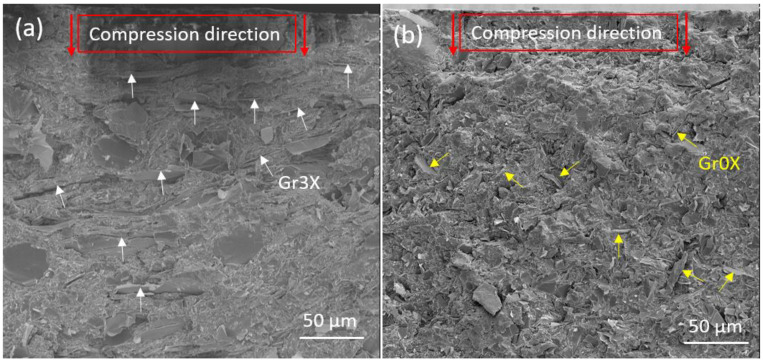
SEM images of the areas close to the top surface of the hot-pressed GnP/polyester composites containing (**a**) 25 wt.% Gr3x versus (**b**) 25 wt.% Gr0x.

For monolithic materials, the lower conductivity values obtained by BDS as compared to the four-probe measurement have typically been attributable to a voltage drop at the interface of the electrode and the sample. In carbon-polymer systems, preferential orientation of the filler induced by the preparation method has also been found to cause this difference. The degree of anisotropy of fillers determines the electrical behavior as a function of direction [25,30,31]. For example, higher in-plane electrical conductivities in GnP/polymer composites prepared by compression molding have been previously reported [32,33]. This behavior is ascribed to the greater compression-induced alignment of filler particles at the areas close to the surface of the sample, as compared to the alignment of the filler particles inside the plate. The same behavior was observed for the samples containing Gr3x, as can be seen in Figure 4a. The microstructure of the polyester/GnPs (Gr3x-25 wt.%) nanocomposite indicates that most of the GnPs are aligned in a direction almost perpendicular to the compression direction. A preferential orientation of GnPs along the surface of the compressed sample was observed for all Gr3x contents that were studied (i.e., 5, 7.5, 10, 15, 20, and 25 wt.%).

The in-plane and cross-plane conductivities of the composite containing 25 wt.% Gr0x measured by four-probe set-up and BDS were 1 × 10^−2^ and 9 × 10^−3^ S/cm, respectively (2 × 10^−1^ and 1 × 10^−2^ S/cm for the sample containing 25 wt.% of Gr3x). As shown in Figure 4b, the composite containing 25 wt.% of Gr0x presents a high degree of anisotropy even in the area close to the surface. Although GnPs at this concentration are close to each other, and individual GnPs cannot be easily distinguished, the random orientation of the platelets is clearly visible.

We observed that in the case of composites filled with larger-particle GnPs, compression molding promotes surface conductivity, with conductivity being nearly one order of magnitude higher than bulk conductivities. According to the SEM images, the compression-induced alignments of GnPs was found to be responsible for this anisotropic behavior. The orientation of the small size GnPs in the composite was not influenced by the compression process as strongly, and consequently, the electrical conductivity of this nanocomposite exhibits only a slight anisotropy.

### 3.2. Thermal Stability

**Figure 5:**
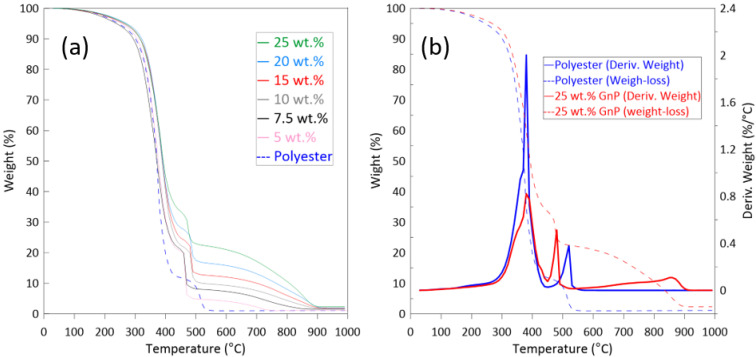
Weight over temperature (**a**) and derivative of weight over temperature (**b**) for neat polyester and polyester filled with Gr3X.

The thermal behaviors of the polyester filled with Gr3X and of neat polyester are presented in Figure 5. The weight loss of the samples as a function of temperature is shown in Figure 5a.

**Figure 6:**
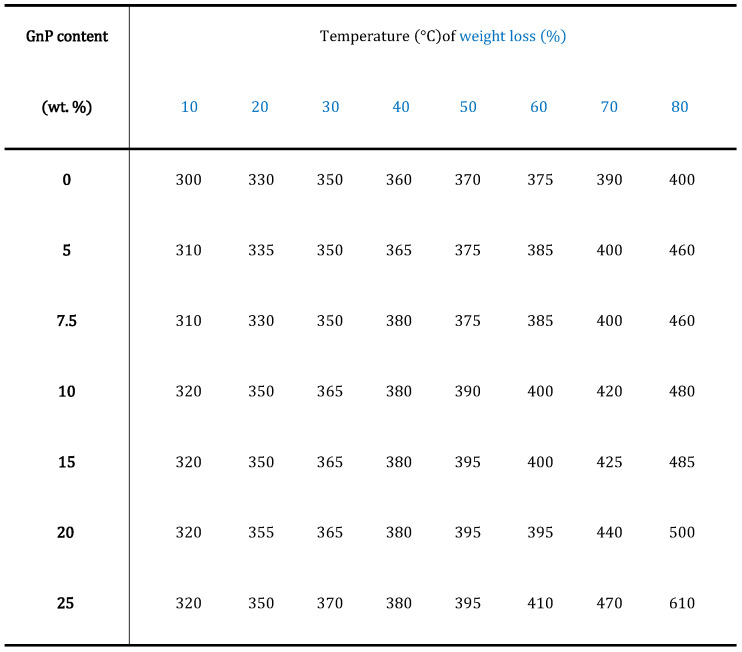
Decomposition temperatures of polyester nanocomposites.

All the filled and unfilled polyester samples were stable up to 200 °C, with no noticeable weight loss. In the case of neat polyester, the first major mass decrease (5.0%) was observed at 230 °C, indicating the loss of moisture. This sample lost 50% of its initial weight by 370 °C, and was completely decomposed (only 1% left) by 550 °C. The first major weight loss of the sample containing 25 wt.% GnP occurred at 270 °C, and this sample kept 50% of its weight until 400 °C. It is not surprising that the GnP-filled polyesters show a slower degradation above 550 °C, since at this stage, it is mainly GnP that is left in the system. Figure 6 indicates the temperatures at which the neat polyester and its nanocomposites lost 10–80% of their initial weight. As can be seen from this table, the thermal degradation process was slightly, but progressively, delayed upon the addition of GnP.

The representatives of the weight derivatives over temperature, seen in Figure 5b, clearly show two principal decomposition steps during TGA under air (oxidative) environment. A similar behavior is reported in the literature [34,35,36]. These two peaks occur in two consecutive stages between the 290 °C and 550 °C temperature range, with the former at the onset of the neat polyester thermal degradation, and the latter at the end of the polyester thermal degradation. Because of the large number of components involved in their formulations, the thermal degradation of thermoset composites is complex. For unsaturated polyester resin systems, the first degradation step is mostly attributable to any volatile degraded compounds (such as phthalic anhydride) and the loss of the cross-linked polyester structure. The second degradation process is more likely a result of random scissions in the polymer chain backbone, as described in the literature [36]. Compared to the neat polyester, the intensity of the main degradation peak is less significant when GnP filler is incorporated into the polyester matrix. The second degradation peak of the nanocomposite material, however, is more intense, and occurs at lower temperatures. In addition to these two major degradation phases, a wide third peak is also observed at higher temperatures for the samples containing GnP, indicating the decomposition of residual GnP char.

### 3.3. EMI Measurements

When the EM wave with an incident energy (*I*) collides with a lossy dispersive material, two waves are created on the surface: a reflected wave (*R*), due to the impedance mismatch between the two mediums, and a transmitted wave into the material [3].

The reflection originates from the interaction of an incoming wave with surface free charges of the shield. In theory, for a transverse EM wave that propagates through a conductive sample ($\sigma \gg \omega \varepsilon_{o}\varepsilon$) with negligible magnetic interaction, the far-field shielding achieved via reflection can be calculated according to the following equation [3,7]:

(4) $$Ref=C_{1}+10\log\frac{\sigma }{2\pi f}$$

where *Ref* is the shielding provided by reflection, *C*_1_ is a constant, *σ* is the electrical conductivity, and *f* is the frequency of the EM wave.

The portion of the EM wave that is not reflected by the shield (*I*-*R*) can be absorbed (*A*) via ohmic loss (by mobile charge carriers) and/or dielectric loss (by dielectric dipoles switching polarizations in alternative EM fields). In the shields with adequate conductivity, the effect of polarization loss is less pronounced than that of mobile charge carriers. In theory, the absorption for such cases is a function of conductivity, and is also proportional to the thickness of the shield [7]:

(5) $$Abs=C_{2}t\sqrt{\pi f\sigma }$$

here *Abs* represents the shielding achieved via absorption, *C_2_* is a constant, *σ* is the electrical conductivity, *f* is the frequency of the EM wave, and *t* is the sample thickness.

It should be emphasized that this approach is not suitable for heterogeneous structures, such as filler-added materials causing a discrepancy between the theoretical equations and the experiments. However, these models have inevitably been used by several researchers to provide estimations and agreements with experimental data involving filler-added materials. The discrepancies are mostly attributable to the following: (1) In partially conducting mediums (imperfect conductor or imperfect dielectric), the dielectric loss is not negligible; (2) the dispersion pattern of the fillers cannot be modeled easily; and (3) the model ignores the multiple internal reflection effect [8,37]. Multiple reflections refer to the reflection process on each plane of the shield, which is typically observed in multilayer structures or filler-loaded systems. Recently, the higher overall shielding efficiency observed for graphene foam nanocomposites as compared to bulk (non-foam) counterparts has been attributable to this multi-reflection mechanism [38,39].

At the same time, measuring the effect of multiple reflections on the overall shielding efficiency cannot be done using currently available measurement techniques. The alternative approach for describing the complex EMI shielding processes of heterogenous structures is to simply represent them by overall reflection and absorption components.

**Figure 7:**
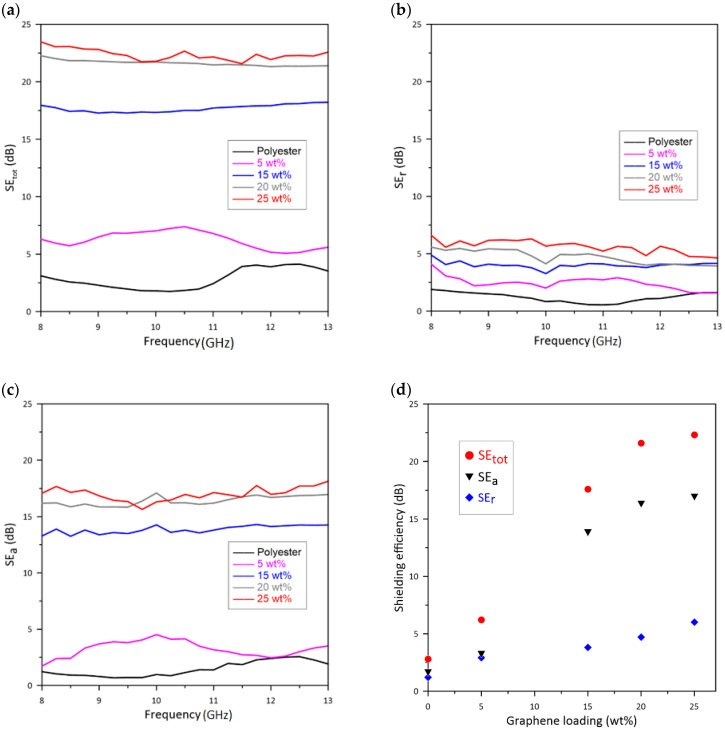
Plots of *SE*tot versus frequency for polyester composites filled with various graphene (Gr3X) contents; (**b**,**c**): *SE*r (**b**) and *SE*a (**c**) values of these polyester composites; (**a**,**d**) average values of *SE*tot, *SE*a, and *SE*r in the X-band frequency range are plotted versus graphene loading.

The amount of the incident EM wave dissipation is described by evaluating the EMI *SE*_tot_, as explained earlier. Figure 7a shows the *SE*_tot_ of the polyester composite disks containing Gr3x fillers over the X-band frequency. Neat polyester has an average *SE*_tot_ of 2.8 dB, and hence, is almost transparent to EM radiation. In contrast, the *SE*_tot_ of the composites increased dramatically as the filler loading increased. For example, the addition of 5 wt.% or 15 wt.% Gr3x increased the average *SE*_tot_ to 6.2 dB and 17.6 dB, respectively. For the sample containing 20 wt.% Gr3x, the average *SE*_tot_ was 21.6 dB, and this level exceeded the target level for commercialization. Meanwhile, the addition of another 5 wt.% Gr3x improved the average *SE*_tot_ of 25 wt.% Gr3x only slightly, to 23 dB.

To investigate the effect of GnP loading on the shielding efficiency, the contributions of the reflection shielding efficiency (*SE*_r_) and absorption shielding efficiency (*SE*_a_) to *SE*_tot_ as a function of GnP loading was examined: *SE_r_* (dB) = −l0 log (1-R) and *SE_a_* (dB) = *SE_tot_* − *SE_r_* [7]. The effect of GnP concentration on *SE*_r_ and *SE*_a_ is shown in Figure 7b,c. Both reflection and absorption were observed to increase as the concentration of Gr3x was increased, and the increase in absorption was more pronounced. While the reflection shielding efficiency of the neat polyester was negligible (average *SE*_r_ = 1.2 dB), it increased to 2.9 dB with the addition of a small loading fraction of graphene (*ϕ* = 5 wt.%). For *ϕ* > 15 wt.% Gr3x (*ϕ* = 20 and 25 wt.%), the increase in *SE*_r_ becomes weaker, and a maximum of ~6 dB was achieved with 25 wt.% Gr3x. The direct relation observed between reflection and graphene loading is due to the greater availability of mobile charge carriers on the surface, at higher concentrations. This behavior is consistent with the theoretical prediction (Equation (1)) for homogeneous conducive materials. A gradual increase in *SE*_r_ at high loadings implies that the formation of a two-dimensional (2D) conductive network of connected GnPs on the surface is completed.

The absorption shielding efficiency of the same composites as a function of EM wave frequency is presented in Figure 7c. The addition of 5 wt.% Gr3x slightly improved the *SE*_a_, but the major enhancement occurred in the samples with 15 wt.% of GnP. After this concentration, the average *SE*_a_ increases slightly and reaches 17 dB for the sample containing 25 wt.% Gr3x. Reinforcing GnP, according to its state of dispersion, distribution, and orientation, can contribute to absorption loss in two ways: (1) by forming a 3D network through cross-plane directions which dissipates the energy of EM wave via ohmic loss, and (2) via graphene or graphene aggregates, which are not connected to conductive pathways. The intrinsic conductivity of these particles acts as independent absorption sites and dissipate the energy of penetrating waves via ohmic loss, interfacial polarization loss, and multiple internal reflection effects.

Comparing the electrical conductivity of the samples containing 20 and 25 wt.% GnP in Figure 1, we can see that the addition of 5 wt.% Gr3x to the composite with 20 wt.% of Gr3x did not lead to an increase in cross-plane conductivity. However, the increase in GnPs concentration from 20 wt.% to 25 wt.% affects the EMI performance of the composite. Therefore, the role of the portion of loaded GnP that did not contribute to conductive pathways should not be neglected in EMI shielding. As evidence, the data in Figure 7b,c show that the composite with GnP fillers below the electrical percolation threshold (*ϕ* = 5 wt.%) still reflects and absorbs EM wave power.

**Figure 8:**
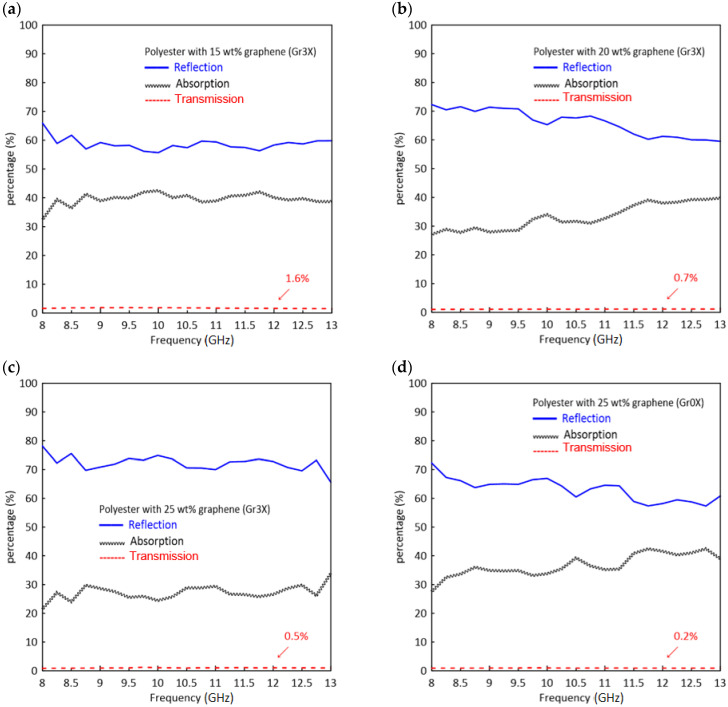
Reflection, absorption, and transmission coefficients of polyester loaded with (**a**) 15 wt.% Gr3x, (**b**) 20 wt.% Gr3x; (**c**) 25 wt.% Gr3x, and (**d**) 25 wt.% Gr0x.

Average values of *SE*_r_, *SE*_a_, and *SE*_tot_ in the X-band frequency range are presented in Figure 8d. At high loadings, the addition of GnP only slightly improved the reflection efficiency of the samples. Meanwhile, the EMI performance of these samples was enhanced because of increased absorption shielding efficiency.

To further examine the data, the power balance [(*A* + *R* + *T*) (%) = 100%] was plotted against the reflection, absorption, and transmission coefficients of the polyester composites loaded with 15 wt.%, 20 wt.%, and 25 wt.% Gr3x filler (Figure 8a–c). For all the compositions, the primary shielding mechanism was reflection, and most of the incident EM waves were immediately reflected (even 4 dB corresponds to 60.2% loss) due to large numbers of charge carriers present at the surface of the samples. As the concentration of GnP filler was increased to 25 wt.%, only 0.5% of the EM wave power was transmitted, while the rest was either reflected (*R* ≈ 74%) or absorbed (*A* ≈ 26%).

**Figure 9:**
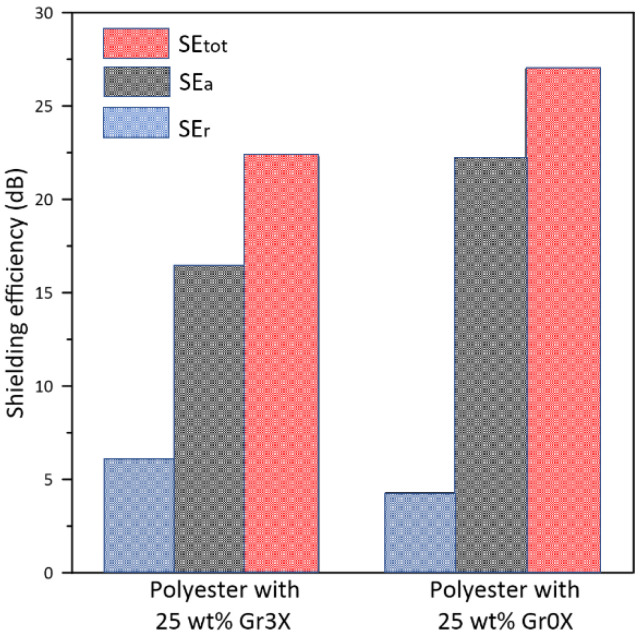
Comparison of the average reflection, absorption, and total shielding efficiency of polyester with 25 wt.% Gr3x versus 25 wt.% Gr0x in the X-band frequency range.

To study the effect of particle size on reflection, absorption, and overall shielding performance, we repeated the measurement with the polyester composites filled with 25 wt.% of the smaller lateral dimension filler, Gr0x. In Figure 9, the average values of *SE*_tot_, *SE*_r_, and *SE*_a_ for 25 wt.% Gr0x were compared with those for 25 wt.% Gr3x.

The composite with Gr3x exhibited a greater reflection efficiency as compared to that with Gr0x. However, the lower reflection of the latter is compensated by a superior *SE_a_* and promoted higher *SE*_tot_ (27 dB). The lower reflection efficiency observed with the Gr0x sample is attributable to the lower in-plane conductivity. It was interesting to observe that the samples had the same volume conductivity, but exhibited significantly different *SE*_a_.

Figure 8d confirms that although reflection is still the major contributor to the shielding mechanism, the composite containing Gr0x reflected less power than did the samples containing Gr3x. For example, the reflection efficiency of the composite with 25 wt.% Gr0x is close to the reflection efficiency of the composite with 20 wt.% Gr3x. This lower reflection indeed helps to increase the contribution of the absorption (*A* ≈ 40%) in the overall EM signal attenuation of these composites.

## 4. Discussion

The primary function of EMI shielding is to reflect radiation using charge carriers that interact directly with the EM fields. However, shielding by reflection cannot fully attenuate or weaken EM radiation energy, and secondary pollution may be caused by the reflected waves. An effective EMI shielding material must both protect the component from external pollution and reduce, or ideally, eliminate undesirable emissions. As a result, shielding materials need to be electrically conductive, but the conductivity is not the only condition. The secondary mechanism of EM shielding requires the absorption of EM radiation. In general, a shield with an ideal EM wave absorbing performance must satisfy two essential requirements: the shielding material should have a good impedance matching with free space to allow the incident EM wave to transmit into the shield, and the material must have a strong EM wave dissipation capacity. The incident microwave energy dissipates within the material through the interactions of the EM field with the material’s molecular and electronic structure, which can convert the EM wave into thermal energy. In recent years, researchers have adopted two strategies to improve the microwave absorption performance of polymer composites:

I Using a combination of magnetic loss materials (such as ferrites) [40], magnetic metals (e.g., Fe, Co, Ni) [7], and dielectric loss carbon materials (such as graphene, CNT) [6];
II Using new complex architectures such as multi-layered gradient structures [41] and foam-based materials [42].

The structural arrangement of conductive fillers in the composite leads to a high likelihood of EMI absorption because of multiple internal reflection effects. Although the proof of concept of these new structures has been previously demonstrated, they usually require additional processing steps and complex nanofabrication, which makes strategy Ⅰ more advantageous over strategy Ⅱ. High magnetic permeability causes a strong natural resonance and eddy current loss between a shielding material and microwaves, and thus, magnetic materials can provide more effective EM wave absorption abilities [7]. However, such materials can hardly exhibit high EMI SE values owing to their low electrical conductivity. Magnetic metals (and their related alloys) generally possess a large saturation magnetization, comparable dielectric loss, and distinguishable permeability in the GHz frequency range, which make them better magnetic candidates for high-performance microwave absorbing materials. Nevertheless, these materials reflect most of the power where, excellent conductivity is generally associated with favorable reflection characteristics. Moreover, large-scale production difficulties resulting from their direction-dependent magnification and high weight penalty are but a few of the other challenges limiting their application.

Among carbon-based nanofillers, graphene represents one of the most studied systems due to its combined light weight and high specific surface area and carrier mobility. Although pure graphene provides excellent shielding against EMI, its absorbing performance is unfortunately not that exciting, due to its relatively high relative complex permittivity and quite low relative complex permeability. To improve the microwave absorption performance, the EM properties of carbon-based composites can be optimized by incorporating secondary magnetic components or dielectric components [5,6,40]. However, many issues still need to be addressed in the ternary, and even quaternary, graphene-based composites, such as difficult dispersion of substrates, poor interfacial compatibility, etc.

In this work, we studied the EMI shielding performance of GnP-polyester composites in the X-band frequency. In particular, we investigated the absorption mechanism of these composites as a function of particle size and demonstrated that, as the size of these fillers is constrained, EM interaction tends to increase. We showed that a sample loaded with a GnP with a larger lateral dimension GnP (Gr3X) exhibits adequate EMI shielding compatibility; nevertheless, this composite suffers from interfacial impedance mismatch due to improper (high) in-plane electrical conductivity. As discussed above, for bulk-shaped composites, the method commonly used to meet the impedance matching requirement involves adjusting the permittivity and permeability values to bring them close to each other (µrεr~1). However, in materials with negligible permeability, reducing the surface conductivity would be the only solution. To reduce the in-plane conduction, we replaced Gr3X with the smaller-size GnP (Gr0X), since the alignment of the GnPs in compression-molded composites could be controlled by the filler’s lateral dimension. Gr0X not only enhanced the impedance matching of the composite, but also led to an improved absorption efficiency (*SE*_a_) as compared to Gr3X. What is not entirely clear, however, is how to explain the dissipation capacity of these composites as a function of particle size.

The main loss mechanisms for nonmagnetic materials are dipolar losses and conduction (ohmic) losses. It should be expected that high electrical conductivity will significantly enhance the imaginary parts of relative complex permittivity, and thus the conduction loss plays the main role. Ionic polarization and electronic polarization can easily be excluded from microwave absorption because they usually occur at a much higher frequency region (10^3^–10^6^ GHz). As discussed in Section 3.3, both composites exhibited a similar cross-plane electrical conductivity, but different EM absorption capacities. Here, we recall the fraction of GnPs which was not a part of the conduction network. When graphene is blended with polymers, the interfacial polarization effect due to microscopic dipoles formation and the formation of a micro/nano capacitive network between the graphene and polymer can significantly attenuate incoming EM waves. Moreover, multiple reflections and interfacial scattering can also occur due to the dielectric constant difference at the interfaces. Therefore, the microwave absorption of these composites originates from the combined effects of conductive loss, polarization, interfacial scattering, and multiple reflection.

The complex permittivity that measures the ability of a material to absorb and store potential electrical energy is a physical property, and is normally related to the structural (particle shape and size) and physicochemical properties. Therefore, microwave absorbers made of materials with different constitutions would have different dielectric values. Furthermore, phenomena such as interfacial polarization, interfacial scattering, and multiple reflections can be enhanced by the decrease in particle size due to an increased surface area.

## 5. Conclusions

Graphene nanoplatelet-filled polyester composites were fabricated through a facile industrial method and their electrical conductivity and EMI shielding performance were studied. To examine the effect of filler geometry on these properties, two grades of GnPs (Gr3x and Gr0x) with different lateral dimensions were employed. The compression-induced GnP orientation was found to lead to anisotropic behaviors of electrical properties in Gr3x composites. In the case of smaller GnPs (Gr0x), the effect of the processing method appeared to be less pronounced.

For the sample containing 25 wt.% Gr3x, an average *SE*_tot_ of 23 dB was obtained. Due to the high in-plane electrical conductivity, these composites reflect almost 74% of the incoming EM waves. Replacing Gr3x with Gr0x enhanced the absorption coefficient to 40%. This characteristic is important for the applications where EMI shielding by absorption mechanism is favored. The transmission coefficient of polyester loaded with 25 wt.% Gr0x was determined to be 0.2%, which corresponds to *SE*_tot_ of 27 dB. These results indicate that the polyester/GnPs composites can be considered as among the most promising candidates for efficient and economic EMI shielding; however, a lot of work remains to be done to satisfy all the technical specifications. Furthermore, future work must be realized to further optimize the morphology of the GnP fillers in order to enhance the absorption coefficient by minimizing the reflection coefficient and thereby obtaining an absorption-dominated shielding enclosure.

## Data Availability

The data presented in this study are available on request from the corresponding author.
